# Gamma-aminobutyric acid interactions with phytohormones and its role in modulating abiotic and biotic stress in plants

**DOI:** 10.1007/s44154-024-00180-y

**Published:** 2024-08-19

**Authors:** Syed Nazar ul Islam, Shaista Kouser, Parveena Hassan, Mohd Asgher, Ali Asghar Shah, Nafees A. Khan

**Affiliations:** 1 Plant Physiology and Biochemistry Laboratory, Department of Botany, School of Biosciences and Biotechnology, Baba Ghulam Shah Badshah University, Rajouri, Jammu and Kashmir 185234 India; 2 School of Biosciences and Biotechnology, Baba Ghulam Shah Badshah University, Rajouri, Jammu and Kashmir 185234 India; 3 Plant Physiology and Biochemistry Laboratory, Department of Botany, Aligarh Muslim University, Aligarh, 202002 India

**Keywords:** Abiotic Stress, Biotic Stress, Gamma-Aminobutyric Acid, Phytohormones

## Abstract

Gamma-aminobutyric acid (GABA), a ubiquitous non-protein 4-carbon amino acid present in both prokaryotic and eukaryotic organisms. It is conventionally recognized as a neurotransmitter in mammals and plays a crucial role in plants. The context of this review centers on the impact of GABA in mitigating abiotic stresses induced by climate change, such as drought, salinity, heat, and heavy metal exposure. Beyond its neurotransmitter role, GABA emerges as a key player in diverse metabolic processes, safeguarding plants against multifaceted abiotic as well as biotic challenges. This comprehensive exploration delves into the GABA biosynthetic pathway, its transport mechanisms, and its intricate interplay with various abiotic stresses. The discussion extends to the nuanced relationship between GABA and phytohormones during abiotic stress acclimation, offering insights into the strategic development of mitigation strategies against these stresses. The delineation of GABA's crosstalk with phytohormones underscores its pivotal role in formulating crucial strategies for abiotic stress alleviation in plants.

## Introduction

Abiotic stress is the negative impact of non-living factors on living organisms in each environment. Common abiotic stresses include drought, salinity, extreme temperatures, heavy metals, cold and ozone etc. (Islam et al. 2023). Abiotic stress primarily affects physiological, biochemical, and cellular alterations in plants (Bhat et al. 2022). It causes necrosis, chlorosis, and decline in photosynthetic efficiency of PS-II (Fv/Fm), pollen sterility and reduction in fruit set. Abiotic stress also alters membrane fluidity and disrupts enzyme activity results retarded growth and crop yield reduction (Atayee and Noori 2020; Bhat et al. 2022). Phytohormones enhance tolerance to abiotic stresses by promoting seed germination, seedling growth, leaf photosynthesis, root growth, and antioxidant enzyme activity. Simultaneously, they mitigate the accumulation of reactive oxygen species (ROS), malonaldehyde, and electrolyte leakage Recent findings underscore the substantial contribution of various phytohormones, such as melatonin (MEL), gamma-aminobutyric acid (GABA), jasmonic acid (JA), salicylic acid (SA), brassinosteroids (BRs), and strigolactones (SLs), to the improvement of abiotic stress tolerance in horticultural plants (Zheng et al. 2023). To avoid these abiotic stresses GABA can help the plants to survive against various environmental stresses (Hasan et al. 2021a, b). Non-protein amino acids constitute a diverse group of nitrogen-containing specialized metabolites widely distributed in the plant kingdom, notably prevalent in various genera within the Fabaceae family. They play essential roles as protective molecules in plants, mitigating oxidative damage and enhancing the tolerance of diverse plant species to various abiotic stresses, such as drought, salinity, and temperature (Rodrigues and Fett-Neto 2019).

GABA, a non-protein amino acid was reported in potato tuber, involved in various physiological processes (Suhel et al. 2022). As a signaling molecule, GABA participates in the regulation of tolerance to various abiotic stresses, such as hypoxia (Yang et al. 2015), salinity (Deinlein et al. 2014), drought (Wu et al. 2016), high temperature (Liu et al. 2019). GABA functions as a key modulator in conferring salt stress resistance by augmenting the antioxidant defense system. It acts as an intrinsic signaling molecule in plant growth and development found in different prokaryotic and eukaryotic species. GABA accumulates and improves plant resilience to abiotic stress through intracellular pH regulation, ion transport, antioxidant system activation, and ROS scavenging. However, research has shown that a specific concentration of GABA can regulate plant growth and the antioxidant system, leading to a reduction in oxidative damage within plants (Li et al. 2017). GABA may act as non-toxic osmolyte and scavenges ROS under the salt stress (Carillo 2018). GABA enhances salt stress tolerance in barley and *Nicotiana sylvestris* with higher content of endogenous GABA and proline (Pro) (Akcay et al. 2012; Ma et al. 2019). It has been reported that exogenously applied GABA regulates osmotic balance in plants and thus enhances stress tolerance without any costly investment (Vijayakumari and Puthur 2016). It has also been shown that under salt stress condition, the production of H_2_O_2_ is inhibited by exogenously applied GABA (Shi et al. 2010a, b). Additionally, it facilitates drought stress resistance by modulating stomatal aperture improves shelf life and storage quality of crop plants (Mekonnen et al. 2016; Xu et al. 2021a, b). Several studies also suggested that exogenously applied GABA protects plants from heat stress such as in *oryza sativa* (Nayyer et al. 2014; Liu et al. 2019). Apart from GABA many other amino acids such as polyamines (PAs), Pro and some other hormones control the stress responses of GABA and regulates the various biochemical reactions by interacting with stress signals (Scholz et al. 2017).

The interplay between GABA and various plant hormones, encompassing abscisic acid, cytokinin, auxins, gibberellins, and ethylene provides strong evidence in expression of stress-responsive genes in aiding plants to cope with a range of stress conditions (Khan et al. 2021; Mishra et al. 2023). In *Citrus sinensis,* the application of GABA resulted in a significant increase in the levels of benzoic acid, cinnamic acid, salicylic acid, trans-jasmonic acid, indole acetic acid, indole propionic acid, and abscisic acid compared to the control (Hijaz et al. 2018). The interaction of GABA with the signaling molecules like Ca^2+^_,_ nitric oxide (NO), hydrogen peroxide (H_2_O_2_) etc. describes the signaling role of GABA in the modification of plant growth and development (Jiao et al. 2019). Thus, the alterations in phytohormone levels during various abiotic stresses coincide with changes in GABA levels, implying potential interactions among multiple players and pathways that contribute to mediating plant stress tolerance. This review provides a comprehensive exploration of GABA biosynthesis, transport and elucidating its intricate cross talk with phytohormones. Emphasis is placed on discerning the multifaceted role of GABA in orchestrating adaptive responses to diverse abiotic/biotic stresses. The intricate molecular mechanisms governing GABA's involvement in stress mitigation are meticulously examined, offering valuable insights into its regulatory functions in the intricate network of plant stress responses.

## GABA Biosynthesis

**Fig. 1:**
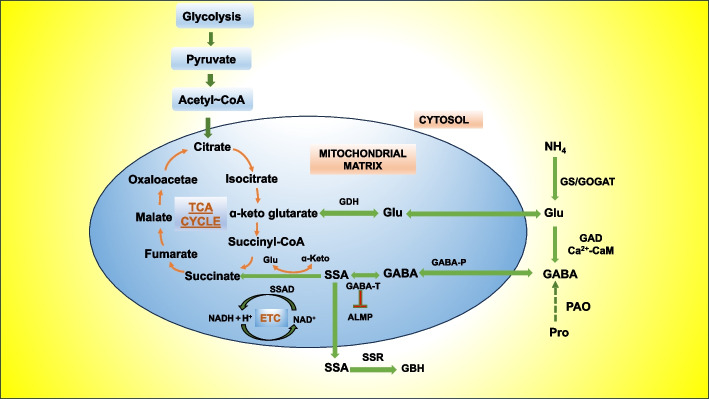
GABA Biosynthesis in plants mediated by GABA shunts. GDH, glutamate decarboxylase; GABA-P, GABA Permease; GABA-T, GABA transaminase; ALMT, aluminum-activated malate transporter; Glu,glutamate;Ala,alanine; Pyr,pyruvate; SSADH, succinic semi-aldehyde dehydrogenase; ETC, electron transport chain; SSR, succinic semi aldehyde reductase; SSA,succinic semi aldehyde; GBH,gamma-hydroxybutyric acid

In plants, the synthesis and metabolism of GABA primarily occur via a branch of the tricarboxylic acid (TCA) cycle known as the GABA shunt, which involves three pivotal enzymes: glutamic acid decarboxylase (GAD), gamma-aminobutyric acid aminotransferase (GABA-T), and succinate semialdehyde dehydrogenase (SSADH) (Studart-Guimaraes et al. 2007). In plants, glutamic acid (Glu) is transformed into GABA by GAD in the cytoplasm. GABA can then enter the mitochondrial matrix through mitochondrial membrane GABA transporters, where it is reversibly converted into succinic semialdehyde by GABA-T (Podlesakova et al. 2019). GAD is a widely distributed enzyme found in both eukaryotes and prokaryotes. It utilizes pyridoxal 5′-phosphate compounds (PLP) and other cofactors to facilitate the specific decarboxylation of intracellular glutamic acid, resulting in the formation of GABA (Khan et al. 2021). As illustrated in Fig. 1, represents the intricate process of GABA biosynthesis in plants, elucidating the enzymatic intricacies within GABA shunts. These pathways orchestrate the conversion of precursor molecules into GABA, providing a visualization of the biochemical events involved in GABA synthesis. Studies have indicated that in plants, the activity of GAD is at its highest when the pH is acidic, with the optimal pH value being 5.8. GAD can modulate its enzyme activity by binding to intracellular Ca^2+^/CaM (Calcium/Calmodulin) when the cell's pH exceeds the normal physiological pH level. Elevated cytosolic Ca^2+^ levels enhance the Ca^2+^/CaM stress response signal, triggering the expression of stress-responsive genes and enzymes in stress-related metabolic pathways. This regulatory mechanism helps maintain proper enzymatic function in response to pH changes in plant cells (Wuddineh et al. 2018).

GABA-T is a vital enzyme found in the cytoplasm, mitochondria, and plastids. It catalyzes the reversible conversion of GABA, transported from the cytoplasm, into succinic semialdehyde within the mitochondria or plastids, playing a pivotal role in GABA metabolism (Clark et al. 2009). Numerous GABA-T variants have been identified in plant species. For instance, GABA-TP in *Arabidopsis thaliana* can utilize pyruvate as an amino receptor, facilitating the transamidation of GABA (Bouché and Fromm 2004). Elevated GAD gene expression due to salt stress triggers extensive GABA synthesis, while mutations in GABA-T prevent GABA degradation, leading to its excess accumulation in plant tissues. This underscores the roles of GABA-T and GAD in plant GABA metabolism during stress responses (Ji et al. 2018; Su et al. 2019). SSADH is a critical enzyme in the GABA pathway and its link to the TCA cycle. Its activity in plants is influenced by NAD^+^ activation and NADH inhibition. A lower NAD^+^/NADH ratio accelerates SSADH activity decline, with near-total loss at a 0.5 ratio. SSADH is also regulated by ATP feedback. Mutations in *Arabidopsis thaliana's* SSADH affect mitochondrial electron transport, indicating its primary function in mitochondria (Bouché et al. 2003; Fait et al. 2005). Various stress conditions can alter cellular NAD^+^/NADH levels by affecting the cell's energy state. This, in turn, can restrict or competitively inhibit SSADH activity, indirectly influencing GABA metabolism (Kilian et al. 2010). A group of membrane-bound transport proteins enables the movement of GABA across cell membranes (Ansari et al. 2021). Glu decarboxylation is not the only way plants produce GABA. It can also be formed through polyamine (PA) degradation, which involves enzymes known as amine oxidases (AOs), including polyamine oxidases (PAO) and diamine oxidases (DAO) (Shelp et al. 2006).

## Transport mechanism of GABA

GABA undergoes transmembrane transport across both plasma and organelle membranes, encompassing intra- and intercellular movement, initially it was identified in animals in 1988 (Bormann, 1988) followed by plants in 1999 (Breitkreuz et al. 1999). Transport plays a crucial role in GABA accumulation and metabolism pathways within cells, with synthesis and catabolism being regulated by various factors, vital for carbon and nitrogen balance maintenance (Yuan et al. 2023). GABA transport within plants encompasses translocation between membranes, including the cell membrane and diverse organelles. This process is modulated by several transporters, including aluminium-activated malate transporters (ALMTs), GABA transporters (GATs), bidirectional amino acid transporters (BATs), and cationic amino acid transporters (CATs). These transporters, situated on the cell membrane or organelle membrane, govern the conveyance of GABA into the intracellular space and various organelles (Meyer et al. 2006; Dündar and Bush 2009; Yuan et al. 2023). GABA can undergo high-rate transport across the cell membrane facilitated by ALMT1, situated on the cell membrane. ALMT1, identified in various plant species including Arabidopsis, wheat, rice, and rape, has been predominantly studied for its role in mediating the transport of GABA, with particular focus on TaALMT1 in wheat (Ramesh et al. 2018). There are twelve homologous genes associated with ALMT found in plants (Ma et al. 2016). Two types of plasma membrane located amino acid transporters, amino acid permease 3 (AtAAP3) and Pro transporter 1,2,3 (AtPROT1,2,3) transport GABA (Breitkreuz et al. 1999). Under diverse stress conditions, GABA accumulation occurs in the cytosol, followed by its transport into the mitochondria for catabolism. The functional characterization of a mitochondrial GABA transporter (AtGABP or GABA-permease) in Arabidopsis has been achieved through complementation in yeast and Arabidopsis gabp mutants (Michaeli et al. 2011). While the influx transporters of GABA in plants have been previously characterized, a recent identification in wheat reveals the existence of a GABA-efflux transporter responsible for transporting GABA from the cytosol to the apoplast (Ramesh et al. 2015, 2017). Plant ALMTs, categorized as anion channels and responsive to various signals, are activated by anions and exhibit negative regulation by GABA (Mora-Macías et al. 2017; Ramesh et al. 2017). Aluminium-activated malic acid transporters (ALMTs) are bidirectional transmembrane anion transporters (Batushansky et al. 2015). Plants possess four homologous genes (GAT1, GAT2, GAT3, and GAT4), with GAT1 situated on the cell membrane facilitating the transmembrane transport of GABA and its translocation from the apoplast to the cytoplasm. In contrast to ALMT1-mediated GABA transport, the presence of Al^3+^ blocks the influx of GABA from the apoplast to the cytoplasm during ALMT1 transport, while it does not affect the GABA transport facilitated by GAT1 (Long et al. 2020). BATs, identified as bidirectional transmembrane transport proteins, are localized on the mitochondrial membrane, Seven homologous BAT genes have been identified in crop species including Arabidopsis, potato, and rice, among BAT1 has got the ability to transport amino acids (Chen et al. 2001; Dündar and Bush 2009; Tian et al. 2020). It was found that AtGABP (At2g01170.1) is a splicing variant of AtBAT1 (At2g01170), which belongs to the APC gene family. This variant primarily plays a role in the transmembrane transport of GABA on the mitochondrial membrane (Michaeli et al. 2011).

Cationic amino acid transporters (CATs) are situated on the vacuolar membrane, and the corresponding CAT gene is affiliated with the APC gene family (Schmidt et al. 2007; Snowden et al. 2015). Nine homologous CAT genes have been identified in plants, with CAT9 playing a predominant role in the bidirectional transport of GABA between the cytoplasm and the vacuole. CAT9 has been recognized in various plant species, including tomato, potato, Arabidopsis, and rice (Whiteman et al. 2008). Within *Arabidopsis thaliana*, GAT1 and GAT2 serve as high-affinity GABA transporters, whereas LAT1 functions as a low-affinity GABA transporter (Meyer et al. 2006). GABA, produced in the cytosol, relies on transporters to accumulate in the vacuole. GAT1 in the plasma membrane aids GABA uptake in root cells under stress, while LAT1 in the tonoplast sequesters GABA into the vacuole (Scimemi 2014).

## Role of GABA in plants to reduce abiotic stress

Gamma-Aminobutyric acid an endogenous modulator plays an important role in plant protection system against various biotic and abiotic stresses (Xu et al. 2021a, b). Abiotic stress promotes GABA buildup via two distinct pathways: Abiotic stress causes metabolic and a mechanical disturbance that leads to cytosolic acidification induces an acidic pH dependent glutamate decarboxylase activation and GABA synthesis. Mei et al. (2016) reported a substantial decrease in cytosolic pH in waterlogged soils which have hypoxia condition and this stress enhanced the accumulation of GABA. Calcium dependent synthesis of GABA in various abiotic stresses such as heat, cold, salinity, drought, and minor environmental fluctuations. These stresses rapidly elevate the cellular levels of Ca^2+^ and increased cytosolic Ca^2+^ stimulates calmodulin-dependent glutamate decarboxylase activity and GABA synthesis (Bhattacharya et al. 2018). The abiotic stress tolerance of plants enhanced by the positive regulation of GABA shunt and associated pathways, whereas, endogenous GABA levels were enhanced by the exogenous application of GABA (Bao et al. 2015; Bhattacharya et al. 2018). In stress situation, GABA is formed by polyamine degradation in plants (Jiao et al. 2019). DAO (Diamine oxidase), a rate limiting enzyme converts putrescine into gamma amino butyraldehyde, further aminoaldehyde dehydrogenase (AMADH) converts gamma-amino butyraldehyde into GABA. Increased movement of high amount of enzyme glutamate decarboxylase and diamine oxidase increases the production of GABA during stress situation (Wang et al. 2017). The GABA quickly accumulates during stress in plants, fungi, cyanobacteria, and bacteria (Wang et al. 2017; Mekonnen et al. 2016).

Drought stress is a predominant factor influencing the growth and development of plant species worldwide (Sehgal et al. 2018). Drought stress is a major challenge in agriculture field that disrupts main physiological processes in plants. Due to long term drought plants suffer from decrease in relative water content, various metabolic and photosynthetic abnormalities. The content of GABA depends upon the plant growth and developmental stage and level of concentrations considerably increased during germination of seeds. Generally, the level of GABA ranges between 0.03 to 2.0 micro mole/gm. Proline, glycinebetaine, and GABA are prevalent compatible solutes, functioning as osmolytes and osmoprotectants. The accumulation of these compounds plays a crucial role in enhancing resistance against various abiotic stresses (Farooq et al. 2009). According to Yong et al. (2017), exogenous administration of GABA is a key strategy for reviving the drought stress in plants. By lowering the level of lipid peroxidation and boosting the photosynthetic pigments and mitochondrial mobility in black pepper, GABA can improve drought resistance (Viyayakumari and Puthur 2016). Exogenous application of GABA eases drought-induced damage in leaves, considerably with higher relative water content; lower electrolyte leakage, lipid peroxidation and leaf wilt (Yong et al. 2017). Exogenous GABA promotes drought-resistance in plants. Furthermore exogenous GABA increases the transaminase and alpha ketone glutarate dehydrogenase activities in white clover leaves (Yong et al. 2017). Application of exogenous GABA to creeping bent grass, which accumulates amino acids, organic acids, and other osmotic materials, also improves drought resistance (Li et al. 2016). Common bean legume nodules had higher GABA concentrations (Serraj et al. 1998). Under drought stress, GABA accumulates in *Glycine max* tissues throughout the plant. However, GAD activities are more prevalent in leaf and root tissues than in nodules because nodules are more permeable to oxygen decline and are therefore more sensitive to oxygen deficiency, which in turn stimulates GAD activities. Thus, exogenous GABA administration may raise the endogenous level of GABA in drought-stressed plants and may enhance their tolerance to drought (Hasan et al. 2021a, b). In addition, exogenous GABA application further activates drought–induced Pyrroline-5-carboxylate synthetase and proline dehydrogenase activities and enhance resistance by modulating stomatal aperture improves shelf life and storage quality of crop plants (Mekonnen et al. 2016; Xu et al. 2021a, b). The activity of the GABA shunt is increased to sustain the TCAC and energy production, though the accurate response depends on the organ (shoot vs. roots) and the stress under consideration (Chen et al. 2019).

Salinity, primarily resulting from NaCl, is a significant abiotic stressor affecting both irrigated and non-irrigated lands. It affects approximately 20% of cultivated areas and nearly 50% of irrigated lands worldwide. Predictions suggest that within the next 25 years, up to 30% of available land may be lost, and this figure could rise to 50% by 2050 due to soil salinity (Wang et al., 2003). According to studies by (Manishankar et al. 2018; Köster et al. 2019), Ca^2+^signalling has been shown to be the main response in plant cells under salt stress. According to Wang et al. (2017) research, exogenously given GABA increases the endogenous synthesis of GABA, which gives resistance against salt tolerance. The photosynthesis and biomass is adversely affected at 150 mM NaCl with an increase in alanine, glutamate and GABA under salt stress (Che-othman et al. 2019). The GABA-T pop2-1 mutant is sensitive to salt, with the exception of osmotic stress, and salt stress increases the expression of genes involved in cell wall and carbon metabolism, primarily sucrose and starch catabolism (Renault et al. 2013). The pop 2–5 mutant over-accumulated GABA in roots and displayed salt tolerance rather than salt sensitivity, according to the results shown for pop 2–1(Su et al. 2019). According to Su et al.'s (2019) findings, GABA causes H^+^ATPase activation, decreases Na^+^ uptake, H_2_O_2_-induced K^+^ efflux, and ROS concentration by employing mutants pop 2–5 and gad 1,2 (with reduced ability of GABA synthesis). The key metabolic enzymes required for the cyclic activity of the TCA cycle were allegedly physio-chemically inhibited by salt in salt-treated wheat leaves, but the increase in GABA shunt activity provides an substitute carbon source for the TCA cycle to function in mitochondria and bypassed salt sensitive enzymes to aid the increase in leaf respiration in wheat plants (Che-Othman et al. 2019).

Further, exogenous GABA increases the endogenous GABA content, activates enzymatic antioxidant activity eases salt damage to plants and enhances salt tolerance in plants when applied to *Zea mays* (Wang et al. 2017), *Trifolium* (Cheng et al. 2018)*, Cucumis melo* (Jin et al. 2019), *Hordeum vulgare* (Ma et al. 2019) and *Solanum lycopersicum* (Wu et al. 2020). GABA accumulation is related to other stresses and hormones in plants in response to salinity. GABA accumulated in durum wheat is combined with high nitrogen or high light treatment under salinity treatment and GABA could also serve as a concise place of nitrogen storage (Carillo 2018). Application of GABA to plants exposed to NaCl affects the production of H_2_O_2_, ABA, and regulate ACS, ACO and production of ethylene in *Caragana intermedia* and Poplar (Shi et al. 2010a, b; Ji et al. 2018). The rapid pace of industrialization, urbanization, intensive agriculture, and mining has led to the extensive pollution of our soil and aquifer resources with heavy metals (Rai et al. 2019). In addition to ion toxicity, heavy metal stress frequently coexists with ROS buildup (Gonzales et al. 2015). According to the metabolite study, GABA content rises in rice roots at 100 µM chromium (Cr) stress (Dubey et al. 2010). It has been reported in soybean treated with 100 µM zinc (Zn) and copper (Cu) stress resulted in high amounts of GABA (Kang et al. 2015). GABA levels are strongly raised in *Nicotiana tabacum* plants treated with moderate (10 µM) Zn concentrations, but are low in plants treated with high (100 µM) Zn concentrations (Das et al. 2016). GABA administration causes the GABA-shunt-related gene expression and activates the antioxidant enzyme system, which confers a tolerance to As (III) in rice seedlings when they are exposed to arsenic (As (III)) stress (Kumar et al. 2017). While under Cd stress, exogenous GABA promoted rhizoid abscission, but Glu addition enhances rhizoid abscission, it appears that endogenous GABA concentration in duckweed is reduced (Yang et al. 2020). According to the studies, metal concentrations may be more closely associated to GABA content than other metal stressors, which do not always result in an increase in GABA content. Furthermore, plant tolerance to metal stress is not always improved by increase in GABA levels (Li et al. 2021).

Cold stress negatively impacts plant growth and development and has the potential to reduce yields by as much as 15–20% (Chaturvedi et al. 2009). The temperature on the earth varies according to the season and day and night. In plants chilling temperature ranges from 0 to 15 °C (Guan et al. 2009; Malekzadeh et al. 2012). Low temperature is a main factor among various factors which affects the yield of plants (Aghdam et al. 2012). Chilling stress is declined by the exogenously applied GABA in peach fruit as reported by Shang et al. 2011. GABA might play a major role in the easing the chilling stress in wheat and by increasing proline content (Malekzadeh et al. 2012). GABA content increases under cold stress (Mazzucotelli et al. 2006; Wang et al. 2014). Exogenously applied GABA also alleviates chilling injury of banana peel and peach fruit during cold storage (Shang et al. 2011; Wang et al. 2014). GABA increases tolerance to chilling stress by enhancing the post-harvest period of various fruits (Aghdam et al. 2015; Malekzadeh et al. 2017).

**Fig. 2:**
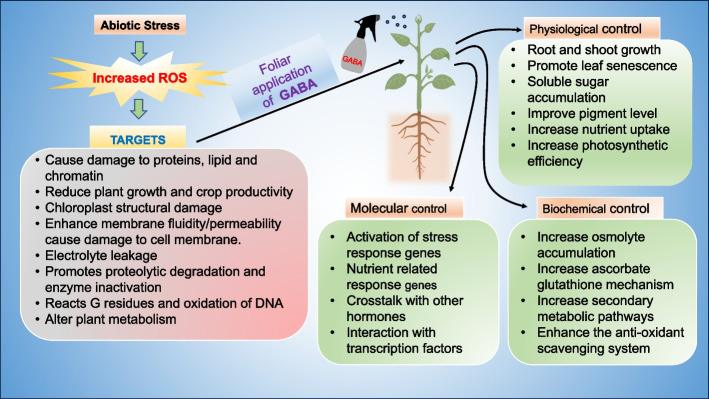
Showing the impact of exogenous application of GABA on physiological, biochemical, and molecular responses in plants

Under diverse abiotic stresses, plants accumulate substantial levels of GABA. This accumulation enables the execution of specific metabolic reactions tailored to the type of stress, contributing to the enhancement of stress resistance in plants (Ramesh et al. 2017). GABA appears to confer partial protection against a spectrum of abiotic stresses in most plants. This protective effect is attributed to mechanisms such as the augmentation of leaf turgor, elevation of osmolyte levels, and mitigation of oxidative damage through the stimulation of antioxidant activities (Sita and Kumar 2020). GABA serves as a non-toxic osmolyte, effectively mitigating ROS and thereby augmenting stress tolerance in plants (Carillo 2018). GABA serves a dual role in plants by not only governing osmotic balance but also influencing the accumulation of H_2_O_2_ and the dynamics of the ascorbic acid–glutathione cycle (Jin et al. 2019). However, it is imperative to engage in further research to elucidate the underlying molecular mechanisms and signaling pathways. These investigations are essential for enhancing crop resilience, optimizing agricultural methodologies, and ultimately advancing global food security. The application of GABA to stressed plants results in elevated GABA shunt activity, enhanced photosynthetic efficiency, increased levels of endogenous GABA, heightened activity of anti-oxidative enzymes, and a reduction in MDA content an oxidative stress marker. This comprehensive response leads to a decrease in reactive oxygen species and ultimately preserves the membrane integrity of the treated plants (Sita and Kumar 2020). Figure 2 Illustrates the comprehensive overview of the effects of exogenously applied GABA on plant systems, encompassing physiological, biochemical, and molecular responses to abiotic stress. Table 1. Showing the application of exogenous GABA manipulates endogenous GABA, antioxidant, phytohormone and secondary pathways, and improves tolerance to abiotic stresses.

**Table 1:** Selected studies to show the role of GABA on some parameters and abiotic stress resilience

**Plant Species**	**Abiotic Stress**	**Biochemical, Molecular and Physiological Responses**	**References**
*Agrostis* *stolonifera*	Drought for 25 d	Increased osmotic adjustment and turf quality, reduced electrolyte leakage. Enhanced chlorophyll and photochemical efficiency	Li et al. (2017)
*Arabidopsis thaliana*	Drought for 7 d	Enhanced stomatal conductance in response to light stimulation and increased water use efficiency	Xu et al. (2021a, b)
*Brassica juncea*	200 µM Cd for 7 d	Increased levels of Pro, GSH, stability of the O_2_-evolving complex, and electron transfer rate in PSII. Enhanced stomatal conductance and expression of P5CS and P5CR. Decreased levels of H_2_O_2_ and MDA	Mahmud et al. (2017)
*Oryza sativa*	30% PEG or/and 150 mM NaCl for 1 week	Increased germination percentage, Vigor index, seedling growth, net photosynthesis, chlorophyll, relative water content Increased K^+^ levels and enhanced activity or expression of GR, SOD, CAT, APX, elevated levels of GABA, sugars, protein, and starch. Decreased Pro, Na^**+**^, H_2_O_2_), O_2_^**•−**^, ^**•**^OH, MDA	Tang et al. (2020)
*Punica granatum*	60 mM NaCl	Enhances the uptake of K^+^, K^+^/Na^+^ ratio, Mg^2+^, P, Mn^+^, Zn, and Fe absorption. Triggering of compatible osmolyte accumulation, encompassing total soluble carbohydrates, starch, glucose, fructose, and sucrose, was observed, acting as an energy source to fortify cellular defense responses against abiotic stress	Zarbakhsh et al. (2023)
*Solanum lycopersicon*	75 mM NaCl for 6 d	Increased growth rate, dry mass and chlorophyll, levels of GABA, Glu, Pro, along with the activation of GAD, SOD, POD, and CAT Decreased levels of ROS, MDA, Na^+^, and reduced Na^+^ flux from roots to leaves	Wu et al. (2020)
*Trifolium repens*	100 mM NaCl for 1 week	Upregulated expression of ZnSOD, MnSOD, FeSOD, CAT, APX, MDHAR. Increased germination percentage, shoot dry matter, and height. Elevated levels of GABA and activity of amylase. Decreased levels of starch, soluble sugars, and amino acids (including Pro). Reduced H_2_O_2_, MDA, and electrolyte leakage	Cheng et al. (2018)
*Triticum aestivum*	100–200 mM NaCl for 21 d; 300 mM NaCl for 24 h	Increased germination, shoot dry matter, chlorophyll, net photosynthesis and activity of SOD CAT. Decreased levels of MDA and electrolyte leakage	Li et al. (2016)
*Vigna radiata*	heat stress (45/28 °C)	Enhanced the reproductive function of plants through enhanced leaf turgor, improved carbon fixation, and assimilation processes, mproved pollen germination, pollen viability, stigma receptivity, and ovule viability	Priya et al. (2019)
*Zea mays*	waterlogging for 14 d	Increased shoot and root dry matter, photosynthetic efficiency, chlorophyll, number of grana per chloroplast, GABA, and activity of SOD, POD, CAT, GR, APX. Decreased levels of MDA, H_2_O_2_, and O_2_^•−^	Salah et al. (2019)

## Role of GABA to modulate biotic stress in plants

Plants adapt physiological and molecular parameters to counter biotic stress, tailoring defense strategies against specific invader (Miller et al. 2017; Asgher et al. 2023). Plants respond to invasion patterns caused by pests or pathogens by activating their defense mechanisms through hormones and complex signaling pathways (Bigeard et al. 2015). Adjusting the metabolic fluxes of soluble sugars and amino acids based on the pathogen's lifestyle is crucial for sustaining strategies that support host defenses or create an unfavourable environment for the pathogen (Seifi et al. 2013a, b). Several primary metabolites play vital roles in plant defense, with GABA standing out due to extensive literature linking it to positive contributions in plant biotic stress as well (Wang et al. 2019). GABA acts as an inhibitory neurotransmitter in vertebrates and is present across Bacteria, Archaea, and Eukaryota kingdoms, exhibiting a diverse range of roles in various organisms (Wu and Sun. 2015; Bown and Shelp. 2016). Throughout plant growth and development, plants encounter various pathogens and pests. In response to biotic stress, there is an observed increase in plant GABA levels (Tarkowski et al. 2020).

Microbe recognition typically initiates pattern-triggered immunity (PTI) as the initial defense mechanism, followed by effector-triggered immunity (ETI). This immune activation involves an early increase in Ca^2+^ influx, leading to the downstream activation of transcription factors that regulate vital immune responses and hormone biosynthesis (Tarkowski et al. 2020). Accumulated GABA can disrupt quorum sensing in response to bacterial pathogens, though some bacteria use GABA as a preferred nutrient source. Additionally, herbivore uptake of GABA can hinder neuronal receptors, leading to neuromuscular disorders, while GABA also enhances endurance by supporting host cells through the TCA cycle and mitigating oxidative damage from ROS bursts (Tarkowski et al. 2020). Glu-dependent GABA shunt activation occurs in response to fungal and bacterial pathogens. The pathogen-induced GABA accumulation is regulated by Ca^2+^ influx resulting from the plant's immune response (Shelp et al. 2006; Tarkowski et al. 2020).

Recognition of pathogens, particularly bacteria and fungi, has been shown to prompt GABA accumulation in several plant species. Suppressing GABA biosynthesis-related genes in tomatoes leads to decreased resistance against *Ralstonia solanacearum.* This downregulation of GABA biosynthesis is anticipated to cause hyper-susceptibility, highlighting the crucial role of GABA in tomato plant resistance to *Ralstonia solanacearum* (Wang et al. 2019). Metabolic fluctuations in response to *Rhizoctonia solani* infection at early and late disease stages in *Glycine max* led to significant increases in transcript and metabolite levels associated with GABA content and ROS signaling (Copley et al. 2017). In incompatible interactions between *Phaseolus vulgaris* and *Phaseolicola*, a notable increase in extracellular GABA accumulation was observed. Leaf inoculation with the avirulent island-carrying strain *Pph* 1302A triggered effector-triggered immunity (ETI), resulting in distinct alterations in apoplast composition. These changes included heightened levels of GABA and other defense molecules, correlating with the initiation of plant defense responses (O’Leary et al. 2016). It was also reported that there is rise in GABA levels when tomato leaves are infected by *Cladosporium fulvum* (Solomon and Oliver 2001). Accumulated GABA in injured plant tissues regulates the expression of the bacterial *attKLM* operon, thereby restraining the quorum sensing of *Agrobacterium tumefaciens* (Chevrot et al. 2006). Elevated GABA shunt activity has recently been linked to enhanced resistance of tomatoes to *Botrytis cinerea* (Seifi et al. 2013a, b). Exposing rice cell suspensions to treatments with *Pyricularia oryzae* lysates resulted in elevated GABA levels, independent of GAD (Forlani et al. 2014). The application of external GABA has demonstrated effectiveness in minimizing symptoms caused by *Botrytis cinerea* on both tomato leaves and fruits (Sun et al. 2019). Also demonstrates a notable reduction in Glomerella leaf spot, lesion lengths and a concurrent increase in antioxidant capacity (Li et al. 2023). Exogenous application of GABA in Arabidopsis resulted in an increase in the activity of enzymes associated with nitrogen metabolism, such as nitrate reductase and GOGAT. This indicates the potential role of GABA not only as a metabolite but also as a signaling molecule, influencing metabolic adjustments in response to necrotrophs (Barbosa et al. 2010).

GABA exhibits intricate connections with primary nitrogen and carbon metabolism, establishing a crucial link with the TCA cycle and playing a pivotal role in the host defenses against diverse pathosystems (Guo et al. 2023). Modulation of biotic stress resistance occurs with endogenous GABA. Specifically, transgenic tobacco and Arabidopsis plants displaying heightened GABA levels demonstrate enhanced resistance to *Agrobacterium* and *Pseudomonas* infections compared to their wild-type plants (Eisenach et al. 2017; Kar et al. 2021). However, tomato plants characterized by exceedingly low GABA levels display heightened susceptibility to *Ralstonia* infection (Chen et al. 2013). The synergistic application of a plant growth-promoting Bacillus sp. strain (JIZ13) and GABA enhances rice growth under salt stress. This is achieved through the regulation of antioxidant enzyme systems, improvement of photosynthesis, and enhancement of soil enzyme activities (Wang et al. 2023). Collectively, these observations strongly substantiate the involvement of GABA in plant defense mechanisms. Thus, regulation of GABA metabolism is emerging as a pivotal mechanism employed by plants to effectively respond to various types of biotic interactions.

## GABA crosstalk with phytohormones under abiotic stress

There are different types of growth regulators called as phytohormones such as auxins, cytokinin (CK), abscisic acid (ABA), gibberellin, and ethylene. These phytohormones have major impact on plant metabolism and play a very important role in the stimulation of plant defense response mechanisms against various stresses. Exogenous phytohormones supplementation has been adopted to improve growth and metabolism under stress conditions. It was reported that the application of ABA and auxin increases GABA production (Kinnersley and Turano 2000). Auxins are among the essential phytohormones and the indole-3-acetic acid (IAA) is the main auxin in plants that regulates the growth and developmental processes such as cell division, elongation, and differentiation (Asgher et al. 2015). The role of IAA/ABA is to regulate the stress of aluminum (Al), as they lead to increase in gene expression of aluminum-activated malate transporters (ALMTs) (Podlesakova et al. 2019). The ALMTs generally do not exhibit activation by Al^3+^, with the exception observed in *Triticum aestivum* (*TaALMTs*). GABA, as indicated by Ramesh et al. (2018), serves as a regulator for these ALMTs. Specifically, GABA acts through ALMTs to negatively regulate malate efflux in plants under Al stress conditions. Additionally, GABA contributes to the regulation of tolerance in plant roots, particularly in response to low pH, high pH, and Al^3+^ ions (Podlesakova et al. 2019). Significant interactions between ABA, IAA, and certain CK derivatives have also been demonstrated to contribute to nitrogen signaling, a component that affects both root and plant growth (Kiba et al. 2011). Thus, it is evident that interference with GABA signaling in various plant processes involved in stress tolerance, root development, nutrient uptake, stress related-ROS response, and metabolism is caused by the crosstalk between auxin and other phytohormones. It is also important to note that the regulation of stress-related responses is influenced by the interactions between phytohormones and GABA, which exhibit both similarities and variations. There are numerous other biological processes that influence plant stress response and tolerance, in addition to the exogenous application of GABA, PAs, and hormones that activate the antioxidative response in many species, but there are many other biological processes that condition plant stress response and tolerance (Bashri and Prasad 2016; Guo et al. 2023). Besides exogenous GABA can well accelerates the PAs synthesis and suppresses the PAs catabolism,which leads to the extremely enhanced different types of PAs content (free Put and Spd, insoluble bound Spd and Spm, soluble conjugated Spd and Spm)under drought stress (Shang et al. 2011).

Cytokinins an important group of plant hormones are responsible for maintaining the cellular proliferation, differentiation, and the prevention of senescence, therefore leading to the inhibition of premature leaf senescence and involved in abiotic stress tolerance (Schmulling 2002; Prasad 2022). Cytokinin levels increases plant stress tolerance through either upregulation of synthesis or deregulation of their degradation. Plants altered with an isopentenyl transferase (IPT) gene, that encodes a CK biosynthetic enzyme, under the control of a stressor senescence-activated promoter (SAG12-ipt) shows improved drought tolerance in rice and cassava (Zhang et al. 2010; Merewitz et al. 2012). A correlation between GABA and CKs can be explained by using the example of barley expressing the CK dehydrogenase 1 gene from Arabidopsis (*AtCKX1*) under the control of a weak root-specific β-glucosidase promoter from maize (Pospíšilová et al. 2016). These transgenic lines over expressed the GABA related gene GAD and ALMT in roots (Pospíšilová et al. 2016). On the other hand, in comparison to the auxin-related stress response, the CK deficient plants shows down regulation of LEA genes and of glyoxylate reductase (GLYR), an enzyme involved in GABA catabolism. When Arabidopsis treated with GABA or with the CK N 6 –benzyl adenine it shows a major decrease in root growth and a high degree of overlap between down regulated and up regulated genes, including those related to sucrose addition and nitrate starvation (Roberts 2007).The crosstalk between GABA and CK regulating stress tolerance has also been explained in tobacco under metal stress, where the zinc tolerance of transformed plants (SAG12 promoter with IPT gene) was associated with accumulation of Pro, methionine and GABA (Pavlíková et al. 2014).

Abscisic acid is a significant phytohormone that aids in regulating several aspects of plant development and growth, including the prevention of fruit ripening and leaf abscission. The "stress hormone," also known as ABA, reacts to a variety of environmental challenges, including both biotic and abiotic stress (Zhang 2014; McQuinn and Waters 2024). Arabidopsis plants that are lack in GABA synthesis shows distorted stomata and impaired stomatal closure (Mekonnen et al. 2016). It has been reported in recent studies that guard cell GABA production is essential for reducing stomatal opening and transpirational water loss by negative regulation of tonoplast localized At-ALMT9 in Arabidopsis (Xu et al. 2021a, b). In *Caragana intermedia,* exogenous GABA has been shown to stimulate the production of ethylene and ABA (Shi et al. 2010a, b; Ji et al. 2018). It was observed that GABA (2 µM) inhibits ABA (2.5 µM) induced stomatal closure at low concentrations and in mutant plants impaired in GABA synthesis (gad2-1) and ALMT9 (almt9), ABA could induce stomatal closure to wild type levels. All these results suggest that there is a cross talk between GABA regulated ALMT9 and ABA in mediating stomatal closure under drought stress (Xu et al. 2021a, b).

Ethylene is considered as a multifunctional phytohormone that regulates both plant growth and senescence. Depending on ethylene concentration and type of plant species it promotes or inhibits the growth and senescence processes. Ethylene is produced from S-adenosyl-methionine (SAM), the activated form of methionine, and the ratelimiting step is the conversion of SAM to 1-aminocyclopropane-1-carboxylic acid (ACC) by ACC synthase. SAM, which is also a precursor in the synthesis of PAs, represents the connection between ethylene and GABA. Moreover, the oxidation of PAs or hydroxylamines and ROS could induce NO production (Wimalasekera et al. 2011), which also controls ethylene production and plant stress tolerance (Manjunatha et al. 2012).GABA increases the expression of ethylene by increasing expression of genes such as ACC synthase and ACC oxidase (ACO) (Shi et al. 2010a, b).A relationship is established between the sequential increase of Ca^2+^, GABA, and ethylene concentrations under stressed conditions (Kinnersley and Turano 2000; Gilliham and Tyerman 2016). By controlling the mRNA level, the GABA controls the synthesis of ethylene (Kinnersley and Turano 2000). In times of NaCl stress, GABA works through ethylene to exert its effects, and because ethylene activity and biosynthesis are inhibited by aminoethoxy vinyl glycine and silver thiosulfate, respectively, GABA limits stem elongation (Shi et al. 2010a, b).

Gibberellic acid is an important plant growth regulator plays an important role in seed dormancy, formation of floral organs and lateral shoot growth (Olszewski et al. 2002; He et al. 2020). In *Cassia italica* high endogenous levels of GA, together with the auxins IAA and IBA, were also related to salt tolerance in GABA treated plants (Alqarawi et al. 2016).Salicylic acid is another important phytohormone having phenolic nature, and its main function is in plant stress tolerance through modulation of antioxidative enzyme activities (Ahmad et al. 2011; da Silva et al. 2017; Spoel and Dong 2024). The mitigation of various abiotic stresses by application of SA was reported by (Senaratna et al. 2000) for water stress by (Azooz et al. 2011) for salt stress and by Ahmad et al. (2011) for heavy metal stress. During stress conditions, SA may have effect on the stress-related metabolites. GABA is a non-protein amino acid that is known for enhancing various stress conditions. Salicylic acid and NaCl enhances the GDH activity, the main role of GDH activity is to convert α-ketoglutarate to glutamate which is the precursor of GABA. Therefore, GDH activity is differently regulated by stress conditions (Akcay et al. 2012). The highest GDH activity was found under salt stress. During single stress conditions, SA might have a role in the accumulation of glutamate which could be then converted to GABA. GAD, the key enzyme in GABA-shunt, catalyzes the conversion of glutamate to GABA. The earlier studies indicate the increase in GAD activity under NaCl stress and supplemental of CaCl_2_ (Yin et al. 2014; Yang et al. 2016). Overall, these observations suggest that phytohormones play a vital role in plant tolerance to various abiotic stresses by modifying the physiological properties and defense system of plants. Studies have demonstrated that ROS are generated in response to abiotic stress. These ROS function as effectors in the downstream stress response, serving as signaling molecules that contribute to the plant's adaptive mechanisms and enhance stress tolerance (Bose et al. 2014). The peroxisomes in plant cells facilitate the basal metabolism of ROS and RNS, resulting in the generation of H_2_O_2_, NO, and GABA through polyamine degradation (Corpas et al. 2019).

**Fig. 3:**
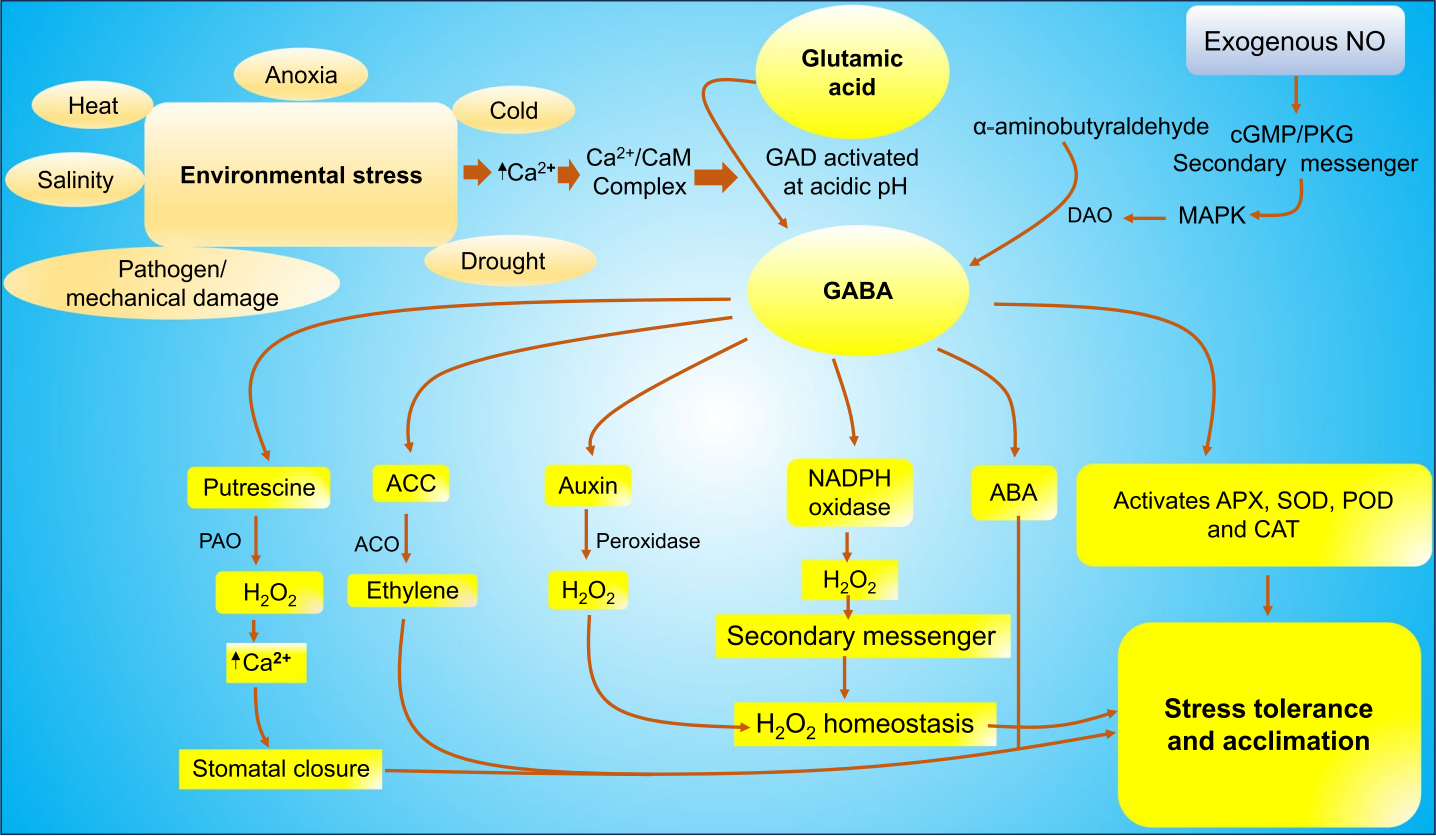
Showing the GABA crosstalk with phytohormones**.** GABA plays pivotal roles in plant stress responses, accumulating rapidly in reaction to drought, heat, cold, anoxia, herbivory damage, and infection, serving as transducers of environmental stress signals. The severity and nature of stress are perceived through alterations in cellular Ca^2+^ levels, activating glutamate decarboxylase (GAD) to produce GABA. Elevated cytosolic Ca^2+^ levels enhance the Ca^2+^/CaM stress response signal, triggering the expression of stress-responsive genes and enzymes in stress-related metabolic pathways. Furthermore, GABA-mediated activation of genes involved in phytohormone biosynthesis elicits physiological stress responses by enhancing antioxidant defenses, modulating H_2_O_2_ levels, and facilitating interactions among phytohormones, and other signalling molecules

Elevated GABA levels under diverse stress conditions suggest its non-utilization by the Krebs cycle, indicating a signaling role. GABA plays a crucial role in pathways associated with ROS elimination, as evidenced by a pronounced increase in ROS levels in mutants lacking GABA shunt gene expression, underscoring the shunt's significance in ROS elimination (Al-Quraan 2015). Thus, the crosstalk between GABA and phytohormones in plants plays a crucial role in modulating various physiological processes and stress responses. Phytohormones interact with GABA to regulate growth, development, and stress tolerance. This intricate interplay influences gene expression, enzymatic activities, and metabolic pathways, ultimately shaping plant responses to environmental stimuli. Further exploration of this crosstalk is essential for deciphering the intricate regulatory networks underlying plant adaptation to changing environmental conditions. Furthermore, GABA-mediated activation of genes involved in phytohormone biosynthesis elicits physiological stress responses by enhancing antioxidant defenses, modulating H_2_O_2_ levels, and facilitating interactions among phytohormones, and other signalling molecules, thereby coordinating abiotic stress responses as illustrated in Fig. 3.

## Interplay of GABA with other signaling molecules

Nitric oxide (NO), a key player in plant stress signal transduction, induces GABA biosynthesis in soybean sprouts under UV-B stress when exogenous SNP, a NO donor, is applied (Li et al. 2019). The mutual impact of GABA on NO production, or conversely, could prove advantageous in enhancing the stress resistance of plants. The exogenous application of an NO inhibitor in conjunction with GABA resulted in an exacerbation of arsenate toxicity. However, the addition of an NO donor alleviated the deleterious effects induced by the NO inhibitor. This highlights the pivotal role of NO in the GABA-mediated arsenate tolerance observed in *Solanum lycopersicum* and *Solanum melongena* seedlings (Suhel et al. 2023). Exposing soybean sprouts to salinity stress revealed that GABA treatment mitigated the suppressive influence of NO on phenolics biosynthesis by enhancing NO production. Conversely, treatment with an NO donor alleviated the inhibitory effect of GABA on phenolics biosynthesis (Xie et al. 2021). GABA supplementation in water-stressed *Trifolium repens* increases NO content by 90%. This reciprocal relationship indicates that NO promotes GABA accumulation, while GABA induces NO accumulation. In soybean sprouts, NO functions as a second messenger, activating gene expression of MAPK via cGMP/PKG, and this activation is facilitated by a transient calcium influx, as reported in tobacco cells (Jiao et al. 2019). Exogenous GABA or NO enhances stress resistance by reinforcing photosynthetic and antioxidant defenses, preserving membrane integrity, regulating osmotic balance and ion homeostasis. This involves upregulating gene and protein expressions and accumulating specific metabolites (Jiang 2023).

Under stress, there was an observed increase in biochemical markers such as H_2_O_2_, MDA, and electrolyte leakage contents, indicative of ROS occurrence. During plant stress, H_2_O_2_ and superoxide radicals serve dual roles as both damaging molecules and signaling molecules (Islam et al. 2023). Concurrently, the exogenous application of GABA demonstrated a mitigating effect by preserving membrane stability through the reduction of lipid peroxidation product formation (Abd Elbar et al. 2021). GABA upregulates enzymes associated with H_2_O_2_ production, including NADPH oxidase, peroxidase, and AO, during stress in *Caragana intermedia*. Exogenous GABA enhances NADPH oxidase gene expression, with a more pronounced effect at 12 h of treatment (Shi et al. 2010a, b). In *Caragana intermedia* under salt stress, elevated peroxidase and AO expression increased H_2_O_2_ production via enhanced peroxisomal enzyme activity due to upregulated gene expression. Exogenous GABA application notably reduced H_2_O_2_ accumulation by attenuating NADPH oxidase activity (Shi et al. 2010a, b). Osmotic stress as well as flooding stress alters cytosolic pH, activating GAD and subsequently elevating GABA production under acidic conditions (Komatsu et al. 2023).

The Ca^2+^-calmodulin pathway is involved in the activation of GAD and the elevation of GABA concentrations, a response observed in diverse range of stresses (Bouché and Fromm 2004). Various stress conditions induce heightened levels of ROS, leading to an increase in cytosolic Ca^2+^ concentration. This is primarily achieved through the opening of Ca^2+^ channels and, subsequently, by the generation of NO, thereby further elevating Ca^2+^ levels (Podlesakova et al. 2019). In abiotic stress, GABA signaling elevates cytosolic Ca^2+^, activating GAD through Ca^2+^-calmodulin. This stress induces upregulation of GABA metabolism enzymes, enhancing GABA levels. GABA enters mitochondria, participating in the TCA cycle, and may modulate gene expression related to signaling and metabolism (Michaeli and Fromm 2015). Elevated cytosolic Ca^2+^ concentration correlates with GABA production and the application of a calcium chelator hindered the gene and protein expression of DAO and AMADH in soybean sprouts. Consequently, this inhibition led to a suppression of GABA production (Jiao et al. 2019). Accumulated GABA mitigates cellular ROS, enhancing plant oxidative stress tolerance. GABA acts as a signaling molecule, impacting pH balance via H^+^-ATPase activation and influencing Ca^2+^ transport through ALMTs, initiating downstream cascades. Knowledge gaps include the polyamine pathway synthesis of GABA and the specific calcium channels activated by GABA, with limited exploration of GABA's signaling role through ROS (Guo et al. 2023). Thus in plants, dynamic alterations in intracellular calcium ion levels govern the activity of the anion channel ALMT12, serving as a receptor for GABA. Consequently, this interaction plays a pivotal role in the modulation of pollen tube growth, implicating the regulatory influence of GABA through its interaction with ALMT12 (Gutermuth et al. 2018).

## Conclusion and future perspectives

In plants, GABA serves as a pivotal regulator of abiotic stress tolerance. Under stress conditions, GABA supplementation enhances GABA shunt activity, augments endogenous GABA levels, and boosts the efficiency of photosynthesis and antioxidative enzymes. Consequently, this leads to a reduction in ROS production, MDA levels, and H_2_O_2_ content, thereby maintain cellular membrane integrity. Key enzymes involved in the GABA shunt pathway, namely GAD, SSADH, and GABA-T play crucial roles in these processes. The expression of genes associated with GABA metabolism undergoes up or downregulation in response to various stressors. Additionally, GABA influences H_2_O_2_ by modulating catalytic activity of many enzymes like NADPH oxidase, prompting further investigation into its role in regulating H_2_O_2_ homeostasis. GABA also interacts with phytohormones and other signaling molecules such as NO and H_2_O_2_ to alleviate oxidative stress. Exogenous application of NO elevates GABA levels, highlighting the potential of GABA-NO signaling in modulating plant growth, development, and stress responses. Nonetheless, gaps persist in understanding the interaction between GABA and phytohormones like salicylic acid and jasmonic acid, representing a frontier in plant biology research. Overall, the positive regulatory role of GABA in plant physiology under adverse environmental conditions suggests that manipulating GABA levels could be a viable strategy for enhancing plant stress resistance. This comprehensive analysis of GABA synthesis and its stress-mitigating functions holds promise for improving agricultural productivity and advancing sustainable development objectives.

## Data Availability

All data generated or analyzed during this study are included in this published article.

## Abbreviations

ABA: Abscisic acid
ACO: ACC-oxidase
ACC: 1-Aminocyclopropane-1-carboxylic acid
ALMTs: Aluminum-activated malate transporters
AMADH: Aminoaldehyde dehydrogenase
APX: Ascorbate peroxidase
CAT: Catalase
cGMP: Cyclic guanosine monophosphate
CK: Cytokinin
CKX: Cytokinin oxidase/dehydrogenase
DAO: Diamine oxidase
GABA: Gamma-aminobutyric acid
GABA-T: Gamma-aminobutyric acid aminotransferase
GAD: Glutamic acid decarboxylase
GBH: Gamma-hydroxybutyric acid
GDH: Glutamate decarboxylase
GLYR: Glyoxylate reductase
IAA: Indole-3-acetic acid
IPT: Isopentenyl transferase
LAT: Low-affinity GABA transporter
MAPK: Mitogen-activated protein kinase
MDA: Malondialdehyde
PAO: Polyamine oxidase.
PKG: Protein kinase-G
POD: Peroxidase
PLP: Pyridoxal 5′-phosphate
ROS: Reactive oxygen species
SAG: Senescence-activated promoter
SAM: S-adenosyl-methionine
SOD: Superoxide dismutase
SSA: Succinic semi-aldehyde
SSADH: Succinic semi-aldehyde dehydrogenase
SSR: Succinic semi-aldehyde reductase
